# Injury incidence and characteristics in adolescent female football players: A systematic review with meta-analysis of prospective studies

**DOI:** 10.5114/biolsport.2024.132996

**Published:** 2024-01-30

**Authors:** Marcos Quintana-Cepedal, Ismael López-Aguado, Ana Fernández-Somoano, Miguel Ángel Rodríguez, Miguel del Valle, Hugo Olmedillas

**Affiliations:** 1 Department of Functional Biology, University of Oviedo, Oviedo, Spain; 2 Asturian Research Group in Performance, Readaptation, Training and Health (ASTURES), University of Oviedo, Oviedo, Spain; 3 Unidad de Epidemiología Molecular del Cáncer, Instituto Universitario de Oncología del Principado de Asturias-Departamento de Medicina, Universidad de Oviedo, Oviedo, Spain; 4 Spanish Consortium for Research on Epidemiology and Public Health (CIBERSP), Madrid, Spain; 5 Department of Cellular Morphology and Biology, University of Oviedo, Oviedo, Spain

**Keywords:** Epidemiology, Football, Adolescent athlete, Injuries, Diagnosis

## Abstract

To observe overall, training, and match injury incidence in female youth football. We also aimed to quantify the incidence of injuries by affected tissue and body location. The following databases were examined: PubMed, Web of Science, Scopus, SPORTDiscus, Cochrane and PEDro. Papers that reported overall injury incidence, training or match injury incidence were included. Additionally, studies had to be performed in adolescent female football players (13–19 years of age). The Newcastle-Ottawa Scale and the checklist of items that must be included in epidemiological football reports were used to assess methodological quality of the included articles. For the meta-analyses, a random effect model was used. A total of 13 studies were included. There were 2,333 injuries; incidence was higher during games (12.7/1000 h) compared to training sessions (2.3/1000 h). The injury match-to-training ratio was 5.8. The lower limbs were the region in which the greatest number of injuries occurred, with the ankle (1.2/1000 h) and knee (0.8/1000 h) having the most injuries. In relation to injured tissue, ligament injuries represented an incidence of 1.3/1000 h, followed by muscle injuries (0.9/1000 h). This study represents the first step towards the creation and implementation of preventative measures in female youth football. The results suggest that attention should be focused on ankle and knee injuries, since they are the most frequent and can lead to sport retirement in some cases depending on the severity.

## INTRODUCTION

Female participation in football is growing in popularity, especially supported by the influence of countries such as France, Sweden, or the United States [1–3]. Nevertheless, as sports participation grows, the incidence of injury increases [4, 5]. This can compromise future physical function and even lead to damage of the professional career [4, 6, 7] or abandonment of sports practice [8, 9].

Since the publication of the Consensus Statement on injury definition and data collection, interest in injury epidemiology in football has risen [10]. Researchers have focused attention on injuries in adult and young footballers, both professional and amateur [11–14]. However, this growth has been significantly biased towards male athletes. Only 21% of the papers included in a recent review reported data on injuries in female adolescent football players [14], high-lighting the underrepresentation of women in research [15]. Another issue in exploring injuries around youth female football is the variability in methodological approaches used between studies.

Differences in injury definition, follow-up time, and study design, among others, are some of the handicaps that prevent researchers drawing strong conclusions regarding this topic [16]. The previous points provide valuable information on what should be the focus when performing a meta-analysis, since the current state-of-the-art only serves to augment uncertainty around decision making.

The existence of anatomical and physiological differences between men and women make imperative the analysis of injuries separately, since this variability influences injury incidence and its characteristics [17, 18]. For example, males sustain more hamstring injuries than female players (0.8 vs. 0.6 injuries per 1000 h of exposure) [19, 20]. On the other hand, females have 2 to 3 times higher risk of sustaining an anterior cruciate ligament (ACL) injury, compared to their male counterparts [21]. Therefore, it seems inappropriate to extrapolate results from one population to another [22]. Regarding age differences, injury incidence in adolescent male football players (5.7/1000 h [14]) is lower than that of male adult players (8.1/1000 h [11]). Main injured locations vary; adolescent injuries affect apophyses and growth plates the most [23], while adult players suffer muscle injuries principally [24].

Previous reviews on young female player injuries have included paediatric participants (inclusion criteria < 19 years of age without a lower threshold), which might confound the results [14]. Also, some criticism has arisen for methodological reasons (i.e. conclusions based on single-point estimates or modification of tools to assess study quality), which implies that results from these investigations must be interpreted with caution [25]. Medical teams prioritize enhancing sports safety by designing and implementing preventive measures, particularly crucial for youth populations. A structured approach, as proposed by van Mechelen, involves three key steps: (1) identifying and describing sport injuries, (2) analysis of risk factors and mechanisms underlying injuries, (3) introduction and effectiveness testing of prevention strategies [26]. We decided to conduct this systematic review with a clear age definition (13 to 19 years of age) and a rigid inclusion criterion due to the variability in injury incidence depending on participants’ age, to address the first step in the “sequence of prevention” [26]. We aimed to summarize the overall information about the incidence, location, type, and severity of injuries in adolescent female football players.

## MATERIALS AND METHODS

The study was registered in PROSPERO (code: CRD42020192263), was conducted in accordance with the methods outlined in the Cochrane Handbook, and is reported according to the PRISMA (Preferred Reporting Items for Systematic Reviews and Meta-Analyses) guidelines [27]. The PRISMA checklist is presented in Appendix A.

### Study selection

The inclusion criteria for studies to be included in this systematic review were established according with the PICOS question (Population, Indicator, Control, Outcome, and Study design) [27]: (P) include adolescent female football players (i.e., players belonging to federated football teams regardless of category and aged 13–19 years), (I) injury definition in terms of time loss (injury that results in a player being unable to take full part in future football training or match play) [10], (C) interventional studies were included when there was a control group which had not undergone an intervention (control group data were utilized), (O) report either overall, training or match injury incidence rate among the surveyed players, (S) have a prospective design. Additionally, a minimum of 11 players (one team) had to be analysed, and articles were required to be written in English and published in a peer-reviewed journal to be included in the review. “Grey literature” (i.e., conference, abstracts, thesis, and unpublished reports) was not taken into account. Any article failing to meet one of the inclusion criteria was excluded.

### Search strategy

**FIG. 1:**
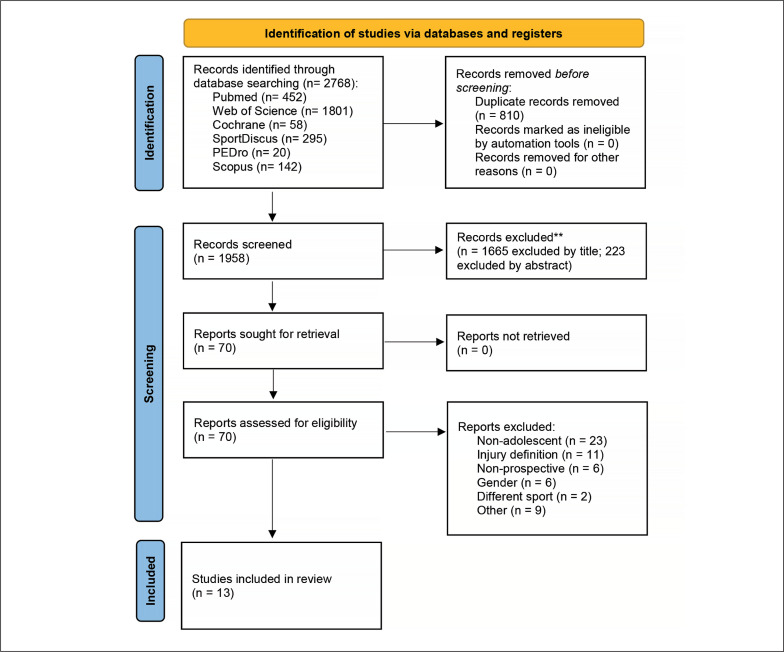
Flowchart of the study selection for overall, training or match injury incidence.

All studies were identified through a systematic search on the electronic databases PubMed/MEDLINE, Web of Science, Scopus, SPORT-Discus, Cochrane and PEDro, from inception until October 3^rd^, 2022. A secondary database search was performed on August 28^th^ 2023, yielding 90 additional records; no additional study was included since inclusion criteria were not met. Reference lists of the included articles were also searched for additional references. Details of the search strategy for PubMed/MEDLINE are shown in Appendix B. Two reviewers (I L-A and HO) independently screened the titles and abstracts of all studies. Duplicate articles were removed using Mendeley Reference Manager. Both reviewers assessed the full text of all potentially eligible articles identified in order to evaluate their possible inclusion in the review. Disagreements over article inclusion were settled through discussion with a third reviewer (MV) until a consensus was reached. The study selection process is presented in Figure 1.

### Data extraction

Data were extracted independently by two reviewers (I L-A and HO) using an electronic data extraction form. The data extracted included the following: (1) General study descriptors (authors, year of publication, place, and study design), (2) characteristics of the subjects (sample size, age and team or place), (3) epidemiological data of injuries (incidence rate and exposure data and distribution by anatomical location). If necessary, the authors of included studies were contacted to provide clarifications or access to raw data. The purpose of the current meta-analysis was to determine the overall effects of: (1) adolescent female football/football-related injury incidence rate (number of injuries×1000 hours of athletic exposure) [28], (2) location of injuries (lower extremity vs. trunk vs. upper extremity vs. head and neck) and (3) affected tissue (muscle vs. ligament).

### Risk of bias and study quality assessment

Risk of bias of included studies was assessed by two reviewers (MQ and HO) independently using a modified version of the Newcastle-Ottawa Scale (NOS). The NOS is a quality assessment tool for cohort and case-control studies which contains eight items categorized into three domains (selection of study groups, comparability of groups and ascertainment exposure) and uses a star rating system to indicate the quality of a study. A description of the items used can be found in Appendix C. The higher the number of stars given to an article, the lower is the risk of bias; studies obtaining > 5 stars were classified as having low risk of bias [12].

The methodological quality of the included studies was evaluated using the “Checklist of items that must be included in epidemiological football reports” following the methodology proposed in a recent systematic review [10]. This checklist consists of 19 items of relevant information that any research in this field should contain; we removed two items (age range and gender of players), since these were part of the inclusion criteria and all the articles met both criteria; the description of each item is available in Appendix D. Any disagreement in the assessment of risk of bias or methodological quality was resolved by mutual consent in consultation with a third reviewer (MV).

### Quality of the evidence

The quality of evidence (QoE) for the meta-analyses performed was graded using the GRADE (Grading of Recommendations Assessment, Development and Evaluation) approach [29]. Initially, it was assumed that the quality was high. Quality was downgraded to moderate, low, or very low when one of the following factors was rated as serious, or very serious: risk of bias, inconsistency, indirectness, or imprecision.

### Statistical analyses

Data analysis was performed by a statistician (AF). Incidence rates (per 1000 hours of exposure) with the corresponding confidence intervals, and number of participants were extracted from the included studies to a Microsoft Excel (Microsoft Excel, Seattle, USA) sheet by one author (MQ). If data were not specifically reported, we calculated them from the available raw data (number of injuries, no. of games or sessions, exposure hours), using the following formulas:

- Incidence = (Ʃ Injuries / Ʃ Exposure) × 1000
- Incidence = no. of injuries / (no. of matches × 11 players × match duration*) × 1000

*Match duration, using the factor 1.5, based on standard 90 min match play [12].

Pooled inverse variance-weighted mean injury incidence rate (IR) estimates along with the 95% confidence interval (CI) for the out-comes were calculated using random effects models based on heterogeneity. The usage of random effects models was decided after applying the Cochran Q test (p < 0.10), which measures betweenstudies heterogeneity. Additionally, the variability between studies was estimated with the τ^2^ statistic. Finally, the statistic I^2^ was used to measure the percentage of total variation in the effect sizes due to heterogeneity, with > 50% and > 75% representing moderate and high heterogeneity, respectively [30]. Sensitivity sub-analyses were performed to manage heterogeneity. Specifically, a leave-one-out influence analysis was chosen, and the estimation did not change meaningfully, supporting the usage of the random effects model. Forest plots were created to illustrate the effects of the different studies and the global estimation of the meta-analysis. The forest plot for each meta-analysis shows the incidence rates of each study and the pooled incidence rate (overall) for the random effect model, all with their 95% CI and on the logarithmic scale. Statistical analyses were performed using Stata 14.2 (Stata Corporation, College Station, TX, USA) and the mar package (V1.4.6.).

## RESULTS

### Study selection

The electronic searches retrieved 2768 references from the literature. After duplicates were removed, 1888 records were excluded during title and/or abstract analysis; 70 full-text copies of the remaining studies were obtained and subjected to further evaluation. At the end of the process, 13 publications met the eligibility criteria and were selected for qualitative and quantitative analysis [1, 2, 31–41].

### Methodological quality assessment

With regards to the risk of bias of the included studies, the mean score obtained on the modified NOS scale was 6 (min: 4, max: 8). Eight studies scored > 5 stars and were considered of low risk of bias [1, 2, 31, 33, 36, 38–40] while the rest received lower scores. The main sources of bias were not reporting the proper methodology of exposure collection or estimating these data (item 4), and the lack of specification of whether the participants were healthy at the beginning of the study (item 5). The mean score for the methodological quality assessment was 16.4 out of 19 possible points [10]. Individual study scores for risk of bias and methodological quality can be found in Table 1.

**TABLE 1:** Characteristics of studies included in the meta-analyses.

Reference	Nº Players	Age	Study type	Exposure (hours)			Nº Injuries			Incidence			Risk of Nº Bias (NOS)	Study Quality

				Overall	Training	Match	Overall	Training	Match	Overall	Training	Match		
Beech et al. 2022 [31]	375	U-16	Elite club season	52,834	46,461	6,374	111	52	59	2.1	1.1	9.3	7	19

Clausen et al. 2014 [32]	438	15 to 18	Club season	27,746	21,461	6,285	269	-	-	9.7	-	-	5	18

Hägglund et al. 2009 [33]	433	17.9 (0.8)	Elite international tournament	1,707	1,210	497	23	9	14	13.5	7.4	28.2	6	18

Le Gall et al. 2008 [1]	119	15 to 19	Elite club season	97,325	87,530	9,795	619	400	219	6.4	4.6	22.4	7	17

Lislevand et al. 2014 [34]	938	13 to 17	Club tournament	-	-	1,318	-	-	8	-	-	6.06	5	15

Schmidt-Olsen et al. 1985 [35]	964	13 to 19	Club tournament	-	-	3,913	-	-	69	-	-	17.6	5	13

Söderman et al. 2001 [2]	175	15.9 (1.2)	Club season	11,689	-	-	79	-	-	6	-	-	7	13

Soligard et al. 2008 [36]	837	15 (0.7)	Club season	45,428	31,086	14,342	212	74	138	4.73	2.38	9.62	8	18

Soligard et al. 2012 [37]	2357	13 to 19	Club season	-	-	18,376	-	-	101	-	-	4.62	5	14

Sprouse et al. 2020 [38]	5852	15 to 19	Elite international tournament	51,151	46,162	4,989	323	190	133	6.31	4.11	26.65	7	16

Steffen et al. 2007 [39]	2020	15.4 (0.8)	Club season	142,721	101,410	41,311	456	113	343	3.68	1.11	8.3	6	18

Steffen et al. 2008 [40]	943	15.4 (0.8)	Club season	65,725	45,869	19,856	241	-	-	3.66	-	-	6	19

Zebis et al. 2018 [41]	185	15 to 18	Club season	-	-	1,559	-	-	29	-	-	18.6	4	15

NOS = Newcastle-Ottawa Scale, U = Under. Age is presented as mean (SD) when possible or as range or category (i.e., U-16), NOS and Study Quality are measured on a 0–8 and 0–19 scale, NOS bold values indicate low risk of bias studies.

### Characteristics of the included studies

Included studies had a prospective cohort design and recorded information on football injuries exclusively. They were published from 1985 [35] to 2022 [31], and the duration of follow-up ranged from 2 days [34] to 8 seasons [1, 38]. The participants’ nationalities were varied; one study was conducted on a Kenyan population [34], one study included participants from international competitions [33], and the rest of the studies were performed in European players [1, 2, 31, 32, 35–40]. Regarding level of play, seven studies were performed on elite/sub-elite players [1, 31, 33, 35, 37, 38, 41], while the rest of the studies collected data on amateur populations. Nine studies reported the overall injury incidence [1, 2, 31–33, 36, 38–40]; six and ten studies reported training [1, 31, 33, 36, 38, 39] and match [1, 31, 33–39] injury incidence, respectively. Regarding secondary outcomes, five studies reported the aggregated lower limb injury incidence [2, 31, 32, 36, 40], two studies reported trunk and head/neck injury incidence [1, 31], six studies reported ankle, knee, thigh, and hip/groin injury incidence [1, 2, 31, 32, 36, 40], three studies reported muscle injury incidence [1, 31, 36], and four reported ligament injury incidence [1, 31, 36, 40].

### Injury incidence

**FIG. 2:**
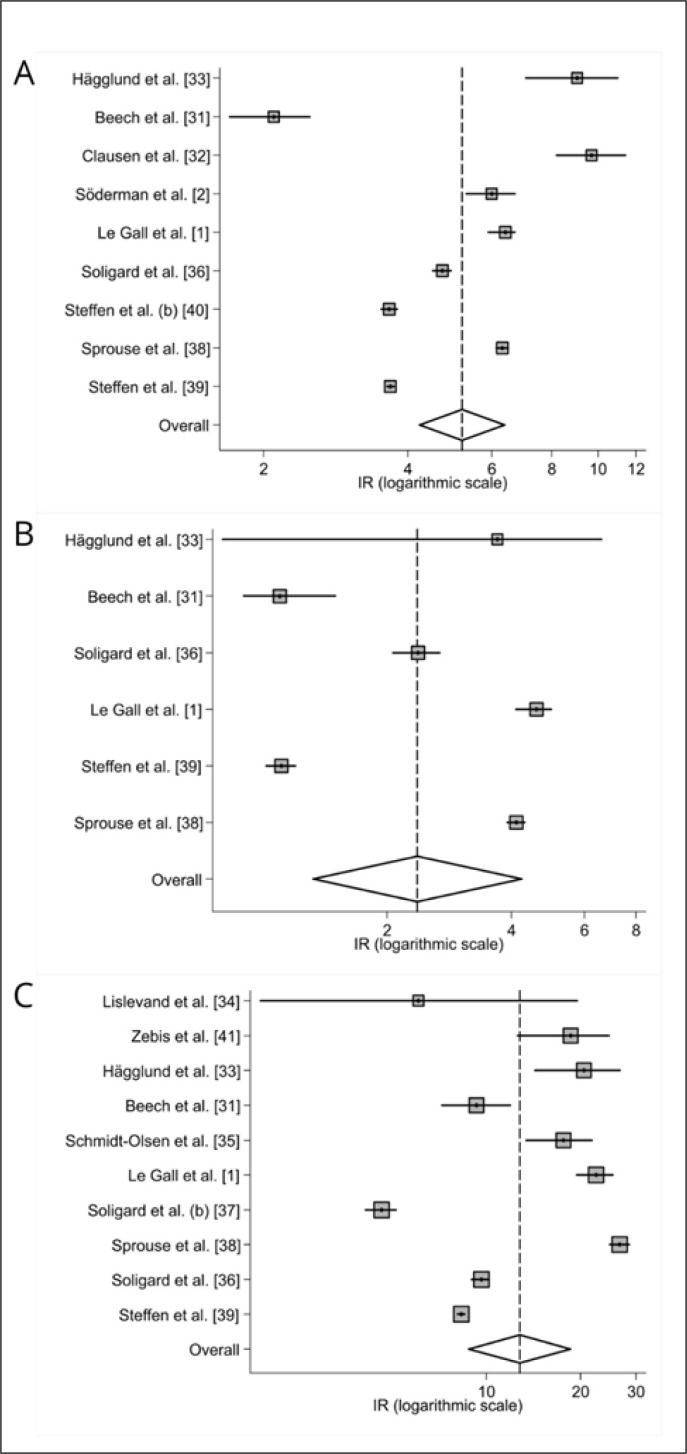
Forest plot displaying overall (A), training (B), and match (C) injuries with the corresponding 95% confidence intervals.

There were 2,333 injuries in total, of which 838 occurred during training sessions and 1,495 were sustained during games. Random effect models showed an overall injury incidence of 5.2 injuries per 1000 hours of exposure (95% CI: 4.2–6.3, Q = 1449.23, p < 0.001; τ^2^ = 0.094; I^2^ = 56%; low QoE), incidence for training injuries was 2.3/1000 h (95% CI: 1.3–4.2, Q = 878.61, p < 0.001; τ^2^ = 0.48; I^2^ = 0%; moderate QoE), and match injury incidence was 12.76/1000 h (95% CI: 8.78-18.53, Q = 1288.09, p < 0.001; τ^2^ = 0.33; I^2^ = 0%; moderate QoE). Injury match-to-training ratio was 5.8. Overall, training, and match injury incidence is displayed in forest plots and can be observed in Figure 2.

### Injury location

Due to the low number of injuries reported in the studies affecting the upper body, trunk, and head/neck, meta-analyses were performed for lower limb injuries. Injury incidence affecting the lower limbs comprised 3.6 injuries per 1000 hours of exposure (95% CI: 2.1–6.4, Q = 1370.29, p < 0.001; τ^2^ = 0.4; I^2^ = 0%; low QoE), secondary analyses for specific body area showed an injury incidence of 0.5 (95% CI: 0.3–0.9, Q = 31.17, p < 0.001; τ^2^ = 0.27; I^2^ = 37.2%; low QoE) (hip/groin); 0.7 (95% CI: 0.5–1.1, Q = 72.6, p < 0.001; τ^2^ = 0.23; I^2^ = 30.6%; low QoE) (thigh); 0.9 (95% CI: 0.5–1.4, Q = 125.84, p < 0.001; τ^2^ = 0.28; I^2^ = 56.2%; very low QoE) (knee); and 1.2 (95% CI: 0.9–1.5, Q = 67.28, p < 0.001; τ^2^ = 0.07;I^2^ = 55.9%; very low QoE) (ankle). Forest plots with 95% confidence intervals for the different locations can be found in Appendix E.

Injuries affecting head/neck had the lowest injury incidence. Beech et al. [31] observed 0.1 injuries per 1000 exposure hours (95% CI: 0.1–0.3), while Le Gall et al. [1] reported an injury incidence of 0.03/1000 h (95% CI: 0–0.1). Injury incidence regarding the trunk rated between 0.04 (0–0.1) and 0.1 (0.1–0.3) injuries per 1000 hours of exposure [1, 31].

Three studies reported upper limb injuries, resulting in a higher injury incidence compared to head/neck or trunk injuries. Steffen et al. [40] observed 0.6/1000 h injuries (95% CI: 0.4–0.7), followed by 0.3/1000 h (95% CI: 0.2–0.4) [1], and 0.2/1000 h (95% CI: 0.1–0.4) [31].

### Injured tissue

Injuries were specified regarding ligament and muscle tissue. Injuries affecting ligamentous structures had an incidence of 1.3/ 1000 hours of exposure (95% CI: 1–1.6, Q = 54.67, p < 0.001; τ^2^ = 0.05; I^2^ = 67.1%; low QoE), while muscle injuries accounted for 0.9 injuries for every 1000 hours of exposure (95% CI: 0.4–1.8, Q = 101.24, p < 0.001; τ^2^ = 0.36; I^2^ = 0%; low QoE). Forest plots with 95% confidence intervals for the different injured tissues can be found in Appendix F.

## DISCUSSION

This systematic review with meta-analysis aimed to quantify injury incidence rates in adolescent female football players. When aiming to mitigate the risk of suffering injuries, the first step is to define the extent of the problem [26]. Previous systematic reviews with metaanalysis have been performed in adults but recently some criticism has arisen due to methodological reasons [25]. This review focuses on this issue, and analyse the topic from a new perspective; included studies had a prospective design, which increases the reliability of the observed results.

### Methodological quality assessment

It is vital to report information in accordance with consensus statements, aiding in the interpretation and analysis of data. We assessed methodological quality with the checklist from Fuller et al. since there is no tool for this purpose in observational studies [10]. In this regard, no threshold was set to determine high-or low-quality studies; it is the authors’ opinion that scores above 15 points (80% of items met) could be categorized as of high quality. Using this threshold, we can be confident that our results are valid, since the mean score of the included papers was 16.4. Additionally, more than half of the included studies were rated as of low risk of bias. Many studies did not report how exposure time was collected; the recording of this value must be accurate since it can affect incidence rates (e.g. when two cohorts have the same number of injuries, the one with lower exposure will have had a higher injury incidence). To properly measure exposure, it is recommended to use weekly forms with individual training and match time.

### Injury incidence

The main findings are that adolescent female football players sustain 5.2 injuries every 1000 hours of exposure, the incidence of match injuries (12.76/1000 h) being considerably higher than the incidence observed for training related injuries (2.37/1000 h). These results are in line with those reported by Horan et al. in female adult players, where overall, training and match injury incidence rates of 5.63/1000 h, 3.27/1000 h, and 19.07/1000 h, respectively, were observed [42]. Two hypotheses are proposed as to why injuries occur more often during matches: (1) Training sessions do not mimic match scenarios; it may be possible that players do not perform match-like actions with sufficient intensity during training. Many injuries happen during explosive actions (sprinting, accelerating, changing directions, or jumping) and players may not be well prepared to tolerate the load that these gestures impose on their muscles and joints [43]. In this regard, exposure to near maximal sprinting speed (> 95% of maximal speed) two days previous to a game can prevent hamstring strains; this effect may also be true for different lower limb injuries [44]. (2) Contact between players may be less common during training sessions; contacting situations can result in injuries that cause long absence from the sport [45]. To account for this point, education and promotion of fair play might be the answer to avoid injuries due to a contact mechanism [46].

### Injury location

Most injuries affected the lower limbs; we observed an injury incidence rate of 3.65/1000 h in this area. Of these lower body injuries, the ankle was the most affected region (1.22/1000 h), followed by the knee (0.89/1000 h), thigh (0.76/1000 h), and hip/groin (0.50/1000 h). Differences were observed compared to male players, who suffer more injuries to the thigh (1.21/1000 h), followed by the ankle (0.91/1000 h) [14]. This can be explained after analysing injuries regarding affected tissue, where females had more ligamentous injuries while males sustained more muscle injuries. It seems therefore that the most common injury in female football is the ankle sprain. The fact that groin injuries had the lowest incidence (0.5/1000 h) is surprising since males have double the incidence of this condition (1/1000 h); these results need to be interpreted with caution, as injury prevalence is similar between genders when non-time-loss groin problems are also considered [47]. Still, anatomical differences in hip and pelvis morphology exist, which may produce differences in the force distribution around the pelvic ring and predispose males to more injuries [18]. However, no clear conclusions can be drawn to date that can explain these differences [48, 49].

### Injured tissue

Our meta-analysis showed an overall time-loss injury incidence rate of 1.30/1000 h and 0.89/1000 h for ligament and muscle tissue, respectively. These results reflect the fact that adolescent players have fewer injuries than adult female elites, who have an injury incidence of 2.7 (muscle) and 2.62 (ligament) injuries per 1000 hours [42]. The difference might due to a constrained calendar in elite football with more games throughout the season and inadequate recovery, favouring the development of lesions [50]. Adolescents face an elevated risk of sustaining ligamentous tears, primarily attributed to the raised concentration of oestrogen during the postmenarchal period, which can affect the integrity of ligament structures, leading to hyperlaxity and increased vulnerability, particularly in the ankle and knee ligaments [51, 52]. Additionally, adolescents undergo a period of significant biological changes, encompassing alterations in muscletendon junctions, ligaments, cartilage, and bone density. These changes may render them more susceptible to injuries due to their reduced capacity to tolerate mechanical loads [53]. Furthermore, during this developmental stage, motor skill performance can be impaired by rapid growth, placing additional stress on tissues beyond their typical limits [54, 55]. Lastly, it must be highlighted that youth players experience heightened exposure to football, characterized by increased session frequency, intensity, duration, diverse game formats, and variations in pitch size. This increased exposure may further augment their susceptibility to injuries [56].

### Future directions

In order to reduce the risk of suffering a certain injury, the first step is to know the extent of the problem (incidence, prevalence, burden); this study aids in that direction but more research should focus on specific injuries such as ankle sprains, anterior cruciate ligament (ACL) tears, or hamstring strains. It is the authors’ opinion that studies on this topic should meet the following criteria to be considered of sufficient quality to be reliable: prospective design, sufficient injury collection time (at least 1 season), diagnosis performed by either a physician or a physiotherapist, regular (weekly or bi-weekly) collection of football exposure, and data report within a clear age frame (i.e., 13–18 years old). Additionally, data on injury-inciting events should be collected using the Football Injury Inciting Circumstances Classification System, since this information adds further value in the prevention sequence and helps in proposing interventions focusing on the exact mechanisms that lead to injuries [57]. Another step could be to differentiate between hip and groin injuries; the last Consensus on football epidemiology suggested collecting these injuries separately [58]. The same principle could be applied to thigh injuries; it is advised to record anterior and posterior thigh injuries separately [19]. Only after we know the exact epidemiological data for these injuries will we be able to implement risk mitigation strategies using randomized controlled trials to test the effectiveness of these measures in reducing the risk of injury. It is of utmost importance to focus on youth populations since most injuries might be easier to sustain after a primer injury has happened already. Implementing programmes is challenging since these athletes tend to get bored or dislike doing extra exercises that demand more time and might lead to delayed onset muscle soreness [59]. Studies on the adoption of prevention programmes have observed that adherence is low [60, 61]; 17–31% of football clubs use hamstring prevention programmes despite its proven effectiveness, and the situation is similar for adductor preventative exercises [60–63]. In this regard, it is advised to educate stakeholders on the benefits of using prevention interventions since effectiveness directly depends on the adherence to the programme.

### Limitations

Several methodological limitations must be acknowledged. Some studies were excluded since the injury reporting or definition was not in accordance with the gold standard proposed by the International Olympic Committee [28], which led to only thirteen studies meeting the eligibility criteria. The same problem arose when collecting certain types of injuries. We originally aimed to incorporate studies with a minimum duration of one season. However, due to the limited number of studies meeting our inclusion criteria, this approach was modified and papers with shorter durations were deemed eligible. This modification may have introduced some bias, as studies ranging from 2 days to 8 seasons were combined in our analysis. The checklist of items that must be included in epidemiological football reports was used, despite not being validated, which could have introduced bias in the quality assessment of the included studies. The quality of the evidence could only attain a moderate level in two of the meta-analyses (training and match injuries), and most results were of low quality due to methodological flaws (low number of studies, heterogeneity, or risk of bias), which downgraded the overall quality of the evidence. Some potentially relevant research may have been overlooked due to the absence of a search in the grey literature. It was not possible to perform quantitative analysis of bone injuries since some articles recorded fractures while others measured other types of bone injuries. Insufficient information on specific injuries could be observed, which inevitably led to reporting aggregated data. Lastly, it was not possible to calculate the injury burden due to the lack of data regarding lay-off days due to injury.

## CONCLUSIONS

Adolescent female football players are exposed to a substantial risk of sustaining injuries. The anatomical region injured most commonly is the lower extremity (knee and ankle) and the most common types of injury are ligament and muscle/tendon. Future studies should focus on introducing and evaluating preventative and training measures that target the most common diagnoses and the results of this review, in order to reduce the number and severity of injuries within female adolescent football players.

## Conflict of interest

The authors declare no conflict of interest.

## APPENDIX A. PRISMA CHECKLIST

**Table:** PRISMA 2020 Checklist

Section and Topic	Item #	Checklist item	Page where item is reported
**Title**			

Title	1	Identify the report as a systematic review.	1

**ABSTRACT**			

Abstract	2	See the PRISMA 2020 for Abstracts checklist.	Not applicable

**INTRODUCTION**			

Rationale	3	Describe the rationale for the review in the context of existing knowledge.	3

Objectives	4	Provide an explicit statement of the objective(s) or question(s) the review addresses.	4

**METHODS**			

Eligibility criteria	5	Specify the inclusion and exclusion criteria for the review and how studies were grouped for the syntheses.	4–6

Information sources	6	Specify all databases, registers, websites, organisations, reference lists and other sources searched or consulted to identify studies. Specify the date when each source was last searched or consulted.	5

Search strategy	7	Present the full search strategies for all databases, registers and websites, including any filters and limits used.	Appendix 2 (Pubmed)

Selection process	8	Specify the methods used to decide whether a study met the inclusion criteria of the review, including how many reviewers screened each record and each report retrieved, whether they worked independently, and if applicable, details of automation tools used in the process.	5

Data collection process	9	Specify the methods used to collect data from reports, including how many reviewers collected data from each report, whether they worked independently, any processes for obtaining or confirming data from study investigators, and if applicable, details of automation tools used in the process.	7

Data items	10a	List and define all outcomes for which data were sought. Specify whether all results that were compatible with each outcome domain in each study were sought (e.g. for all measures, time points, analyses), and if not, the methods used to decide which results to collect.	7

	10b	List and define all other variables for which data were sought (e.g. participant and intervention characteristics, funding sources). Describe any assumptions made about any missing or unclear information.	7

Study risk of bias assessment	11	Specify the methods used to assess risk of bias in the included studies, including details of the tool(s) used, how many reviewers assessed each study and whether they worked independently, and if applicable, details of automation tools used in the process.	7–8

Effect measures	12	Specify for each outcome the effect measure(s) (e.g. risk ratio, mean difference) used in the synthesis or presentation of results.	8

Synthesis methods	13a	Describe the processes used to decide which studies were eligible for each synthesis (e.g. tabulating the study intervention characteristics and comparing against the planned groups for each synthesis (item #5)).	8

	13b	Describe any methods required to prepare the data for presentation or synthesis, such as handling of missing summary statistics, or data conversions.	8

	13c	Describe any methods used to tabulate or visually display results of individual studies and syntheses.	Not applicable

	13d	Describe any methods used to synthesize results and provide a rationale for the choice(s). If meta-analysis was performed, describe the model(s), method(s) to identify the presence and extent of statistical heterogeneity, and software package(s) used.	8

	13e	Describe any methods used to explore possible causes of heterogeneity among study results (e.g. subgroup analysis, meta-regression).	8

	13f	Describe any sensitivity analyses conducted to assess robustness of the synthesized results.	8

Reporting bias assessment	14	Describe any methods used to assess risk of bias due to missing results in a synthesis (arising from reporting biases).	Not applicable

Certainty assessment	15	Describe any methods used to assess certainty (or confidence) in the body of evidence for an outcome.	Not applicable

**Table:** PRISMA 2020 Checklist

Section and Topic	Item #	Checklist item	Location where item is reported
**RESULTS**			

Study selection	16a	Describe the results of the search and selection process, from the number of records identified in the search to the number of studies included in the review, ideally using a flow diagram.	6

	16b	Cite studies that might appear to meet the inclusion criteria, but which were excluded, and explain why they were excluded.	Figure 1

Study characteristics	17	Cite each included study and present its characteristics.	Table 2

Risk of bias in studies	18	Present assessments of risk of bias for each included study.	9, 11

Results of individual studies	19	For all outcomes, present, for each study: (a) summary statistics for each group (where appropriate) and (b) an effect estimate and its precision (e.g. confidence/credible interval), ideally using structured tables or plots.	12–13

Results of syntheses	20a	For each synthesis, briefly summarise the characteristics and risk of bias among contributing studies.

	20b	Present results of all statistical syntheses conducted. If meta-analysis was done, present for each the summary estimate and its precision (e.g. confidence/credible interval) and measures of statistical heterogeneity. If comparing groups, describe the direction of the effect.	12–13

	20c	Present results of all investigations of possible causes of heterogeneity among study results.	14

	20d	Present results of all sensitivity analyses conducted to assess the robustness of the synthesized results.	8

Reporting biases	21	Present assessments of risk of bias due to missing results (arising from reporting biases) for each synthesis assessed.	Not applicable

Certainty of evidence	22	Present assessments of certainty (or confidence) in the body of evidence for each outcome assessed.	Not applicable

**DISCUSSION**			

Discussion	23a	Provide a general interpretation of the results in the context of other evidence.	13–15

	23b	Discuss any limitations of the evidence included in the review.	16

	23c	Discuss any limitations of the review processes used.	16

	23d	Discuss implications of the results for practice, policy, and future research.	15–16

**OTHER INFORMATION**			

Registration and protocol	24a	Provide registration information for the review, including register name and registration number, or state that the review was not registered. 4 (blinded)

	24b	Indicate where the review protocol can be accessed, or state that a protocol was not prepared.	4

	24c	Describe and explain any amendments to information provided at registration or in the protocol.	14

Support	25	Describe sources of financial or non-financial support for the review, and the role of the funders or sponsors in the review.	None

Competing interests	26	Declare any competing interests of review authors.	None declared

Availability of data, code and other materials	27	Report which of the following are publicly available and where they can be found: template data collection forms; data extracted from included studies; data used for all analyses; analytic code; any other materials used in the review.	None

From: Page MJ, McKenzie JE, Bossuyt PM, Boutron I, Hoffmann TC, Mulrow CD, et al. The PRISMA 2020 statement: an updated guideline for reporting systematic reviews. BMJ 2021;372:n71. doi:10.1136/bmj.n71. For more information, visit: http://www.prismastatement.org/

## APPENDIX B. SEARCH STRATEGY FOR PUBMED/MEDLINE

**Table:**

	PubMed/MEDLINE
#1	“Soccer”[Mesh] OR “football”[Mesh]
#2	“Soccer”[tiab] OR “football”[tiab] NOT “American football”[tiab] NOT “rugby”[tiab]
#3	#1 OR #2
#4	“Injuries”[Mesh] OR “Injury” [Mesh]
#5	“Injuries”[tiab] OR “Injury” [tiab]
#6	#4 OR #5
#7	“Prevalence”[Mesh] OR “Incidence”[Mesh]
#8	“Prevalence”[tiab] OR “Incidence”[tiab]
#9	#7 OR #8
#10	“Female”[Mesh] OR “Women”[Mesh]
#11	“Female”[tiab] OR “Women”[tiab]
#12	#10 OR #11
#13	#3 AND #6
#14	#9 AND #12
#15	#13 AND #14
#16	Filters: Adolescent: 13–18 years, Female

## APPENDIX C. RISK OF BIAS ASSESSED WITH THE NEWCASTLE-OTAWA SCALE (NOS)

**Table:**

NOS	1	2	3	4	5	6	7	8	Total
Beech et al. 2022	*	*	*	*		*	*	*	7
Clausen et al. 2014	*	*	*				*	*	5
Hägglund et al.2009	*	*	*	*		*		*	6
Le Gall et al. 2008	*	*	*		*	*	*	*	7
Lislevand et al. 2014	*	*	*			*		*	5
Schmidt-Olsen et al. 1985	*	*	*			*		*	5
Söderman et al. 2001	*	*	*		*	*	*	*	7
Soligard et al. 2008	*	*	*	*	*	*	*	*	8
Soligard et al. 2012	*	*	*			*		*	5
Sprouse et al. 2020	*	*	*	*		*	*	*	7
Steffen et al. 2007	*	*	*		*		*	*	6
Steffen et al. 2008	*	*	*		*		*	*	6
Zebis et al. 2018	*	*	*				*		4

Modified Newcastle-Otawa Scale item description (extracted from López-Valenciano et al. 2021).

**Table:**

Item		Description
1.	Description of Sport and participants	There are several types of football players (sub-elite vs. elite, males vs. females). Without the description regarding to the type of football players it is impossible to conclude which population the incidence rates refer to. Studies that reported a description of the football players or informed the type of football players receive a star for this criterion. Studies conducted in football tournaments (which may determine the type of football players; e.g., World Cup tournaments) and which describe the race characteristics receive a star for this criterion as well. Studies that did not describe the characteristics or the type of football players, and studies conducted in football tournaments that did not describe the characteristics of the tournament did not receive a star for this criterion.

2.	Injury definition	Studies that aimed to investigate football-related injuries should present a definition of an injury informing what was considered as an injury in the study. Studies that present a definition of time-loss injury received a star for this criterion.

3.	Representativeness of the exposed cohort	(a) Truly representative of the average football players in the community*; (b) somewhat representative of the average football players in the community*; (c) selected group of users; (d) no description of the derivation of the cohort.

4.	Exposure time collection	(a) Secure record*; (b) structured interview*; (c) written self-report; (d) exposure was estimated, (e) no description

5.	Demonstration that the outcome of interest was not present at the start of the study	(a) Yes*; (b) no. Studies that described that all football players included were injury-free at baseline received a star for this criterion.

6.	Assessment of Outcome	(a) Independent blind assessment*; (b) record linkage*; (c) self-report; (d) no description.

7.	Follow-up time for outcomes to occur	(a) Yes*; (b) no. Studies that carried out a follow-up period of at least 12 weeks received a star for this criterion.

8.	Adequacy of cohorts follow-up	(a) Complete follow-up of all subjects accounted for*; (b) subjects lost to follow-up unlikely to introduce bias (up to 20% loss) or description provided of those lost*; (c) follow-up rate < 80% and no description of those lost; (d) no statement. A loss to follow–up greater than 20% may increase the risk of bias in prospective studies (Fewtrell et al., 2008).

* Articles with this alternative received a star for this criterion.

## APPENDIX D. CHECKLIST OF THE METHODOLOGICAL QUALITY ANALYSIS

**Table:**

	1	2	3	4	5	6	7	8	9	10	11	12	13	14	15	16	17	Ʃ^*^
Beech et al 2022	✓	✓	✓	✓	✓	✓	✓	✓	✓	✓	✓	✓	✓	✓	✓	✓	✓	19
Clausen et al 2014	✓	✓	✓	✓	✓	✓	✓		✓	✓	✓	✓	✓	✓	✓	✓	✓	18
Hägglund et al 2009	✓	✓	✓	✓	✓	✓	✓	✓	✓		✓	✓	✓	✓	✓	✓	✓	18
Le Gall et al 2008	✓	✓	✓	✓		✓	✓	✓	✓		✓	✓	✓	✓	✓	✓	✓	17
Lislevand et al 2014	✓	✓	✓	✓	✓	✓	✓		✓		✓	✓	✓	✓	✓			15
Schmidt-Olsen et al 1985	✓	✓	✓	✓	✓	✓	✓	✓				✓		✓	✓			13
Söderman et al 2001	✓	✓	✓	✓	✓	✓		✓				✓	✓		✓		✓	13
Soligard et al 2008	✓	✓	✓	✓	✓	✓		✓	✓	✓	✓	✓	✓	✓	✓	✓	✓	18
Soligard et al 2012	✓	✓	✓	✓		✓	✓		✓		✓	✓	✓	✓	✓			14
Sprouse et al 2020	✓	✓	✓	✓	✓		✓	✓			✓	✓	✓	✓	✓	✓	✓	16
Steffen et al 2007	✓	✓	✓	✓	✓	✓	✓	✓	✓	✓		✓	✓	✓	✓	✓	✓	18
Steffen et al 2008	✓	✓	✓	✓	✓	✓	✓	✓	✓	✓	✓	✓	✓	✓	✓	✓	✓	19
Zebis et al 2018	✓	✓	✓	✓	✓	✓	✓		✓	✓		✓	✓	✓	✓			15

Checklist: Study design (1)Duration (2) Organisational setting (3) Location (4) Nº Teams (5) Nº Players (6) Level of play (7) Medical personnel involved (8) Injury recording frequency (9) Exposure data recording frequency (10) Training to improve quality of data collection (11) Injury definition (12) Exposure definition (13) Nº of match exposures (14) Nº of match injuries (15) Nº of training exposures (16) Nº of training injuries (17).

* All the included studies reported age and gender. Therefore, both items are summed to the final score.
